# 1189. COVID 19 Infection in Children in Colombia, Experience from a Nationwide Network (CORONARED)

**DOI:** 10.1093/ofid/ofab466.1381

**Published:** 2021-12-04

**Authors:** Juan Gonzalo Mesa-Monsalve, Ivan Felipe Gutiérrez Tobar, Alejandro Diaz Diaz, Juan Pablo Calle-Giraldo, Yamile K Chaucanez-Bastidas, Juan Francisco López-Cubillos, Laura Mendoza-Rosado, Patrik Eliana Sarmiento-Wilches, Luis M Sosa-Ávila, Luis Fernando Mejía-Rivera, Juan P Rojas -Hernandez, Catalina Arango-ferreira, Álvaro Darío Hoyos-Orrego, Diana Cristina Ortiz-Marín, Rosalba Vivas-Trochez, Catalina Jaramillo-Arango, carlos Garces, Eduardo López Medina, Luis Gabriel Vinasco-Sánchez, Paula Araque-Muñoz, Juan Pablo Londono-Ruiz, Claudia Patricia Beltrán-Arroyave, Isabel C Hurtado-Palacios

**Affiliations:** 1 Hospital General de Medellin/Clínica Las Américas Auna, Envigado, Antioquia, Colombia; 2 Clinica Infantil Colsubsidio, Clínica Infantil Santa María del Lago, Bogotá, Distrito Capital de Bogota, Colombia; 3 Hospital General de Medellin, Medellin, Antioquia, Colombia; 4 Clínica Versalles/Clínica Farallones/Clínica Palma Real/Clínica occidente, Cali, Valle del Cauca, Colombia; 5 Hospital Infantil Los Ángeles, Pasto, Narino, Colombia; 6 Fundación HOMI, Bogota, Distrito Capital de Bogota, Colombia; 7 Clínica Laura Daniela, Valledupar, Cesar, Colombia; 8 Clínica Materno Infantil San Luis, Bucaramanga, Santander, Colombia; 9 Universidad Industrial de Santander / Clínica materno infantil San Luis, Bucaramanga, Santander, Colombia; 10 Fundación Clínica Infantil Noel, Cali, Valle del Cauca, Colombia; 11 Club Noel Children’s Hospital, Cali, Valle del Cauca, Colombia; 12 Hospital Universitario San Vicente Fundación/Universidad de Antioquia, Medellín, Antioquia, Colombia; 13 Clínica Universitaria Bolivariana/Universidad Pontificia Bolivariana, Medellín, Antioquia, Colombia; 14 None, Medellín, Antioquia, Colombia; 15 Clínica Soma/Procaren, Medellín, Antioquia, Colombia; 16 E.S.E Hospital Manuel Uribe Ángel, Medellín, Antioquia, Colombia; 17 Universidad de Antioquia, Medellin, Antioquia, Colombia; 18 Centro Médico Imbanaco, Cali, Valle del Cauca, Colombia; 19 Universidad Tecnológica de Pereira, Pereira, Risaralda, Colombia; 20 Clínica la Colina, Bogota, Distrito Capital de Bogota, Colombia; 21 Universidad El Bosque, Bogotá, Distrito Capital de Bogota, Colombia; 22 Universidad de Antioquia, Clinica El Rosario, Clinica del Prado, Medellin, Antioquia, Colombia; 23 Universidad del Valle, Cali, Valle del Cauca, Colombia

## Abstract

**Background:**

Worldwide SARS-CoV-2 infections increase every day. Despite the infection is less severe in children, it can be severe and associated with complications. However, local data remain scarce. We sought to describe epidemiological and clinical characteristics of COVID-19 infection in this population across different age groups.

**Methods:**

Observational, multicenter study across 23 Colombian hospitals from 22 different territories. We included all patients from 0 months to 17 years with confirmed SARS-CoV-2 infection by either antigen or RT-PCR testing.

**Results:**

From March 1, 2020, to October 31, 2021, we identified 1,186 patients: neonates (88), 1 to 3 months (130), 4 to 23 months (306), 2 to 4 years (169), 5 to 11 years (229) and 12 to 18 years (226) with confirmed COVID-19 infection. Of those,77(6.2%) were asymptomatic, 631(53.2%) hospitalized, 132(11.2%) required PICU. 58 cases met WHO definition of MIS-C. Patients less than 24 months of age were characterized by fever (74%) and more respiratory distress (30.1%) compared to other groups. Patients >5yo seemed to have a more severe presentation. They had more gastrointestinal (GI) symptoms (31% vs 37.8%), had more need for ICU care given presentation with shock increased with age ( >5yo 9.5%; 5-12yo 10.6%; 12-18yo 11.5%). Lab markers including thrombocytopenia and Lymphopenia were more common on this age group. Antibiotic treatment was common (%%) especially in neonates (40.9%), despite bacterial coinfection was rare (8.7%), length of hospitalization was longer in older than 2-year-old groups. 23(1.9%) patients died, similar across different age groups.

Heat map by age group

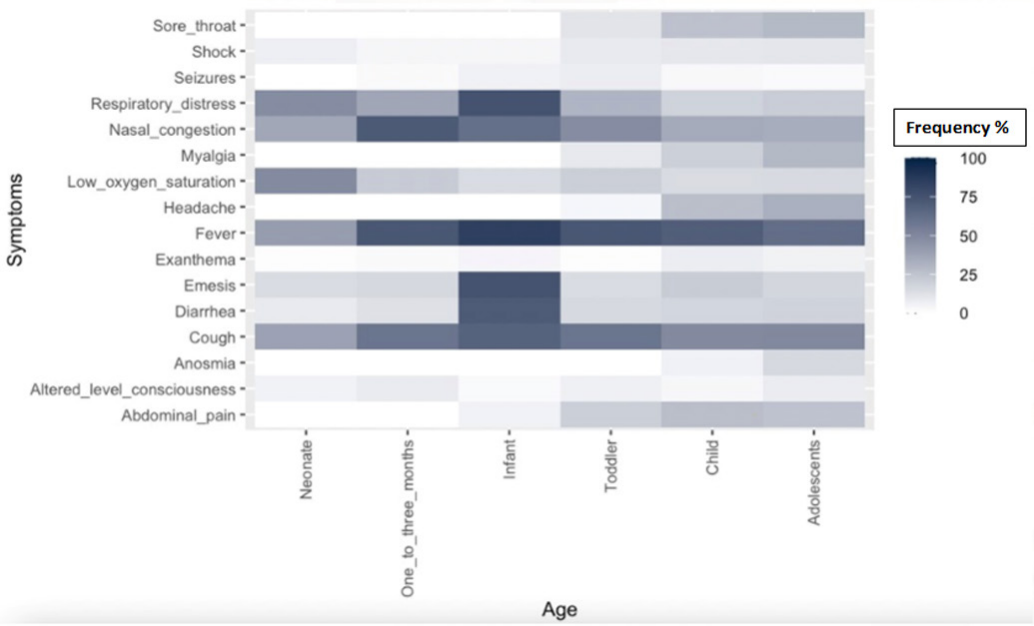

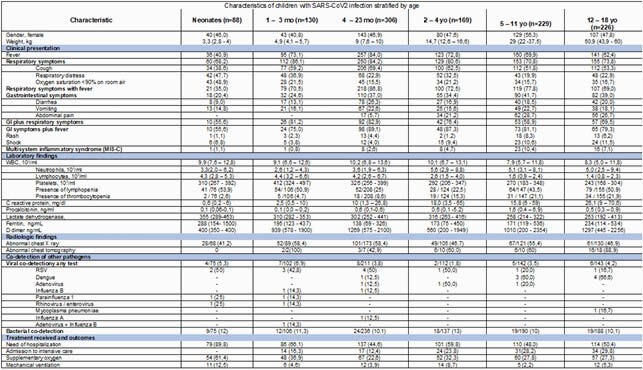

**Conclusion:**

COVID-19 infection in Colombian children presented differently across different age groups. Children older than 5 years had a more severe clinical course and prolonged hospital stays. Clinical findings according to age groups could help clinicians in characterizing and identifying COVID 19 infections in Children.

**Disclosures:**

**Ivan Felipe Gutiérrez Tobar, n/a**, **Pfizer and MSD** (Advisor or Review Panel member, Research Grant or Support, Speaker’s Bureau, Has received support from Pfizer and MSD for participation in congresses and has received conference payments from Pfizer)**Pfizer and MSD** (Speaker’s Bureau, Other Financial or Material Support, Has received support from Pfizer for participation in congresses) **Juan P. Rojas -Hernandez, Candidate for doctorate in Public Health**, **Pfizer** (Other Financial or Material Support, Has received support from Pfizer for participation in congresses) **Eduardo López Medina, n/a**, **Pfizer** (Other Financial or Material Support, Has received support from Pfizer for participation in congresses)

